# Screw fixation of ACPHT acetabular fractures offers sufficient biomechanical stability when compared to standard buttress plate fixation

**DOI:** 10.1186/s12891-019-2422-6

**Published:** 2019-01-24

**Authors:** Tatjana Busuttil, Michel Teuben, Roman Pfeifer, Paolo Cinelli, Hans-Christoph Pape, Georg Osterhoff

**Affiliations:** 1 Department of Trauma, University Hospital Zurich, Zurich, Switzerland; 2 Department of Orthopaedic, Trauma and Plastic Surgery, University Hospital Leipzig, Liebigstrasse 20, 04103 Leipzig, Germany

**Keywords:** Acetabular fracture, Acetabulum, Pelvis, Osteoporosis, Biomechanical screw fixation, plate fixation

## Abstract

**Background:**

Geriatric acetabular fractures require fixation with sufficient primary stability to allow for immediate full-weight bearing. Minimally-invasive procedures would be desirable in order to keep perioperative morbidity low. The purpose of this study was to compare the biomechanical strength of lag screw-only fixation of anterior column posterior hemi-transverse (ACPHT) acetabular fractures to standard anatomical plate fixation.

**Methods:**

Standardized ACPHT fractures were created in fourth generation synthetic pelvis models and stabilized by either an anatomical buttress plate (*n* = 4) or by a screw-only construct (n = 4). In a validated setup, a cyclic loading protocol was applied with increasing axial force (3200 cycles, 175 N to 2250 N). Construct survival, acetabular fracture motion, and mode of failure were assessed.

**Results:**

The median number of cycles needed until failure of the construct occurred was 2304 cycles (range, 2020 to 2675) in the plate fixation group and 3200 cycles (range, 3101 to 3200) for the screw fixation constructs (*p* = .003). With regard to energy absorbed until failure, the plate fixation group resisted to 1.57 × 10^6^ N*cycles (range, 1.21 × 10^6^ to 2.14 × 10^6^) and the screw fixation group to 3.17 × 10^6^ N*cycles (range, 2.92 × 10^6^ to 3.17 × 10^6^; *p* = .001). All plate fixation specimens failed with a break-out of the posterior-column screw in the quadrilateral wing of the anatomical plate within a maximum load of 1750 N while the screw fixation constructs all survived loading of at least 2100 N. Acetabular fracture gap motion, acetabular rim angle, and medial femoral head subluxation as measures of fracture displacement were all not different between the two groups (*p* > 0.1).

**Conclusions:**

In this in vitro biomechanical study, screw-only fixation of an ACPHT acetabular fracture resulted in at least as good construct survival as seen for standard buttress plate fixation. Both methods resisted sufficiently to forces that would be expected under physiologic conditions.

**Electronic supplementary material:**

The online version of this article (10.1186/s12891-019-2422-6) contains supplementary material, which is available to authorized users.

## Background

Demographic changes in industrialized countries come along with an increase in fragility fractures [1]. The combination of impaired bone quality and a loss of postural stability frequently [2, 3] results in geriatric low-energy fractures also of the acetabulum, typically Judet and Letournel type anterior column posterior hemitransverse fractures (ACPHT) [4].

While the general principles of articular fracture reduction and fixation also apply to fragility fractures of the acetabulum, the goals can be different with injuries in the young. The aim is to restore the function of the hip joint, allow pain-free early mobilization to reduce the complications of bed rest, decrease early mortality and minimize the risk of post-traumatic arthritis. However, most geriatric patients may not be able to follow a postoperative partial weight-bearing regimen [5] and immobilization over more than a few days is associated with increased in-hospital complications [6] and mortality [7]. Hence, it is important to achieve an early and stable fixation to allow for immediate full weight bearing [8]. Second, surgical interventions too invasive may be associated with increased perioperative morbidity and mortality in elderly patients. Especially with open procedures, acetabular fractures in the elderly are associated with noticeable post-operative complication rates [9]. Thus, instead of open reduction and plate fixation, percutaneous procedures using screw-only constructs have been advocated [6, 10, 11].

There is much debate, however, about the standard fixation of ACPHT fractures and whether screw fixation alone is biomechanically stable enough in order to allow for immediate postoperative full-weight bearing. While there are biomechanical studies on the strength of different acetabular plate fixation constructs, only few address type ACPHT fractures [12, 13] and – to the authors’ knowledge – a direct comparison of plate and screw fixation is yet missing.

Hence, the purpose of this study was to compare the biomechanical strength of a lag screw-only fixation to a standard anatomical plate fixation of an ACPHT acetabular fracture in an in vitro experimental setup.

## Methods

### Specimens

Eight fourth-generation composite hemipelves (Sawbones; Pacific Research Laboratories, Vashon, WA, USA) were used for this biomechanical investigation.

**Fig. 1:**
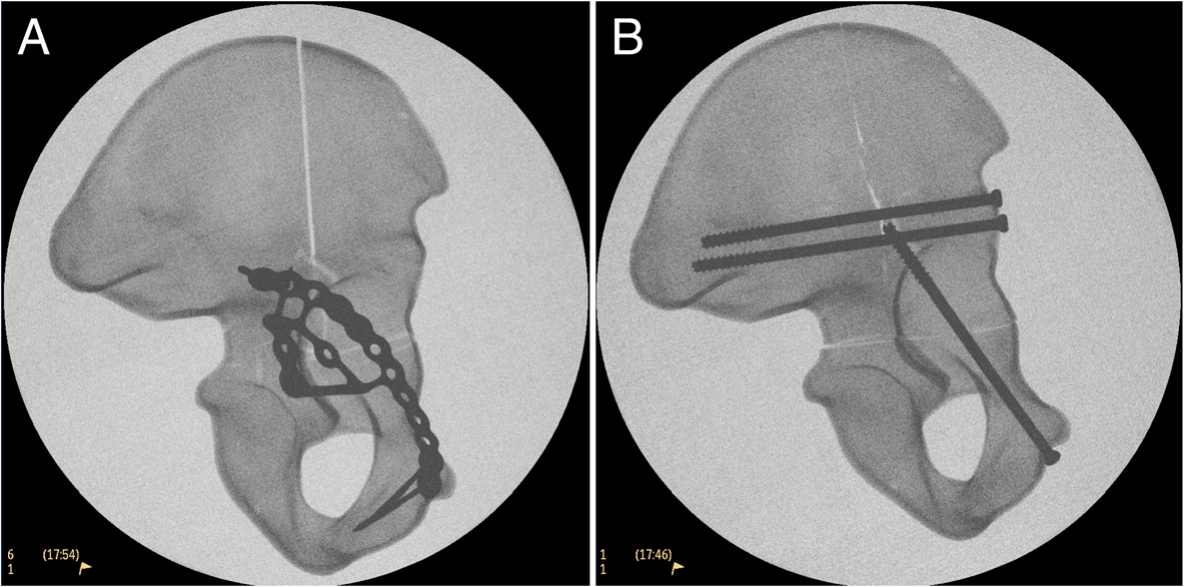
Testing samples. In the plate fixation group (**a**) an anatomically pre-shaped plate with a quadrilateral buttress was applied and fixed to the three fragments of a standardized ACPHT acetabular fracture using 3.5-cortical screws. In the screw fixation group (**b**), fracture configuration fixed using two 6.5 mm-cannulated LC2-screws and one 6.5 mm-cannulated retrograde anterior column screw

Standardized left-sided acetabular fractures, Judet & Letournel type anterior column posterior hemi-transverse (ACPHT), were created and the specimens were randomly assigned to either screw or plate fixation (Fig. 1 A + B):

In the plate fixation group (*n* = 4), the fracture was reduced and an anatomical acetabular steel plate with quadrilateral buttress was applied (PRO Suprapectineal Plate, Stryker, Selzach, Switzerland) and fixed with seven 3.5 mm bicortical screws (at least two screws in each of the three fragments).

In the screw fixation group (n = 4), the fracture was reduced and one cannulated steel screw (6.5 mm, 32 mm-partially-threaded; DePuySynthes, West Chester, PA, USA) was inserted in terms of a retrograde anterior column screw and another two screws were placed along the supraacetabular canal in terms of two LC2-screws [10, 14].

### Biomechanical testing

**Fig. 2:**
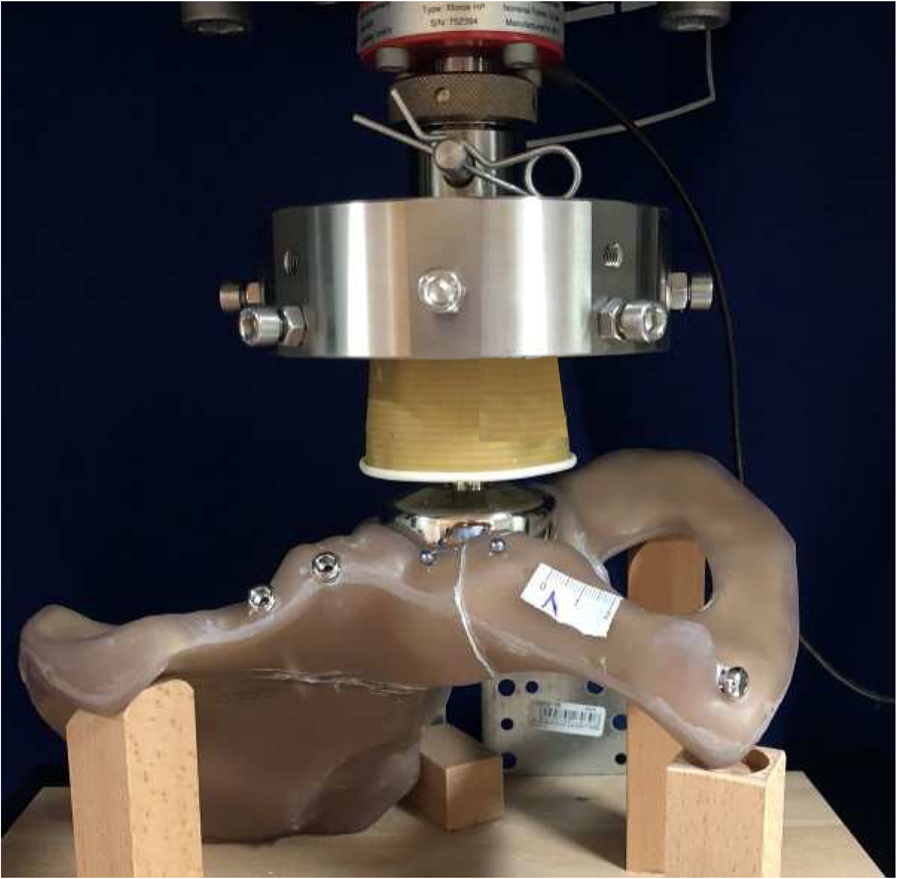
Testing setup. On an universal testing machine cyclic loadings were applied on the acetabular joint surface through an endoprosthetic femoral head that was linked to a load cell

The experimental setup was designed to provoke implant failure or loosening of the screws by fatigue or damage accumulation in the area of the bone-implant interface [15]. The samples underwent mechanical testing of a previously described setup [16–18] using a universal testing machine (Zwick GmbH & Co, Ulm, Germany. Fig. 2). Each specimen was subjected to consecutive cyclic loadings at 1 Hz beginning with a sinusoidal axial force from 17.5 to 170 N. After every 500 cycles, the axial load was increased to simulate higher walking stresses (partial weight-bearing, full weight-bearing, climbing steps, stumbling) up to 1750 N and after that every 100 cycles up to 2250 N (Table 1). The force was applied in medio-superior direction through an endoprosthetic femoral head component on the left acetabulum [16]. Both ilium and pubic symphysis were allowed to freely rotate on the testing setup. Grease was used to decrease friction between the load applicator and the acetabular joint surface. Before testing, all specimens were pre-loaded with 175 N to level out initial subsidence.

**Table 1:** Cyclic loading protocol

Cycles	Load range (N)	
	Min	Max
0–500	17.5	175
501–1000	35	350
1001–1500	70	700
1501–2000	105	1050
2001–2500	140	1400
2501–3000	175	1750
3001–3100	210	2100
3101–3200	245	2450

Visual markers were attached to the pelvis models prior to testing and digital imaging was obtained to allow for later opto-metric analysis.

Construct failure was defined as either a sudden loss of force resistance over 30% or visual breackage of the model or femoral head dislocation resulting in an immediate stop of testing.

### Outcome variables

**Fig. 3:**
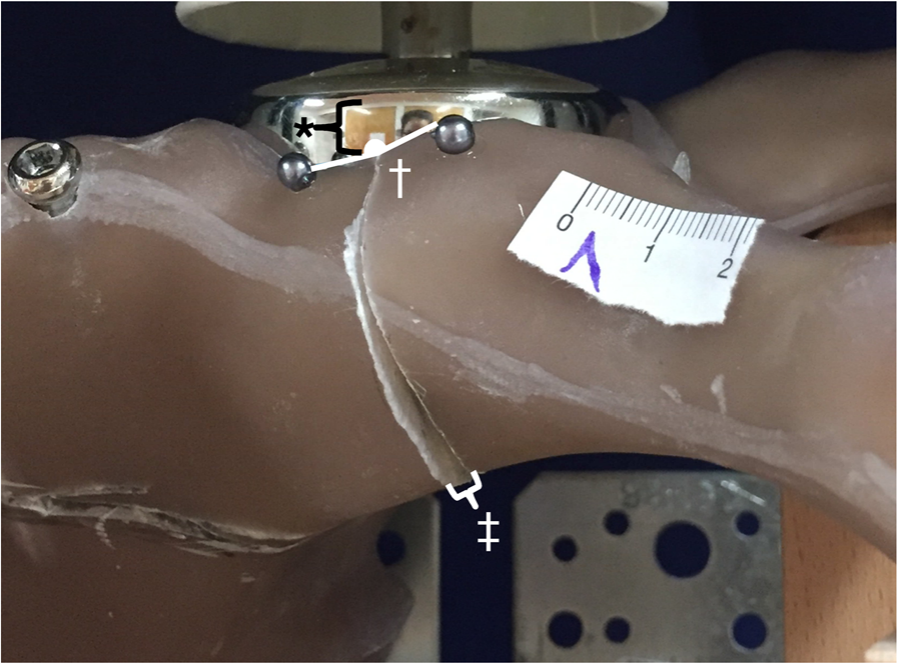
Measurement of fracture displacement. As measures of fracture displacement, the acetabular fracture gap (‡), the acetabular rim angle (†), and the medial femoral head subluxation (*) were measured under loading with 175 N every between every series of cycles

(1) Primary outcome parameter was *construct survival,* measured in cycles [n] to failure (as defined above) and in energy absorbed by the construct [N*cycles], defined as the number of cycles to failure multiplied with the force each cycle was applied with.

(2) Secondary outcome parameters were measures of acetabular fracture motion and were assessed opto-metrically between every series of cycles under loading with 175 N by the use of the calibration and caliper tool of ImageJ2 [19], including

- *acetabular fracture gap* [mm], defined as width of the interfragmentary intrapelvic cortical gap just above the dome (Fig. 3),
- *acetabular rim angle* [°], defined as the angle between the pubic and iliac part of the anterior acetabular rim, and
- *medial femoral head subluxation* [mm], defined as the change in distance between the acetabular rim and the endoprosthetic femoral head component’s center of rotation.

In case of construct failure, parameters of fracture displacement were assessed one last time under loading with 175 N. Hence, data was obtained only for the first five rounds of cyclic loading.

(3) An additional descriptive outcome parameter was the *mode of failure*.

### Statistical analysis

Primary outcome was construct survival. It was our hypothesis that screw fixation provides comparable stability as plate fixation within the load ranges of partial weight-bearing (maximum 2.0 body weights [20]), but not beyond this. Hence, with an anticipated non-inferiority margin of 200′000 N*cycles, assuming a standard deviation of ±10% and with a significance level of *α* = 0.05 and a desired power of 0.80, a sample size calculation revealed a minimum sample size of 4 per group [21].

Post-test analysis was done using SPSS for Windows V25.0 (IBM, Chicago, IL, USA). All data is reported as medians with the minimum and maximum value (range). Parametric tests were used to compare differences between the two groups. The level of significance was defined as *p* < 0.05.

## Results

### Construct survival

**Fig. 4:**
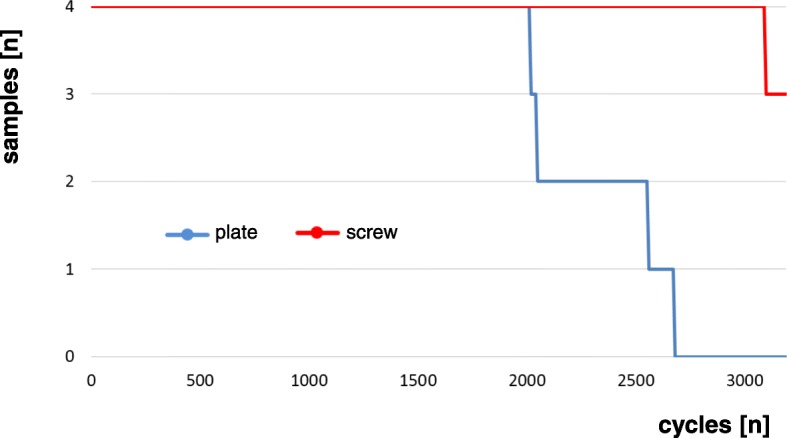
Construct survival

The median number of cycles needed until failure of the construct occurred was 2304 cycles (range 2020 to 2675) in the plate fixation group and 3200 cycles (range, 3101 to 3200) for the screw fixation constructs (*p* = .003, Fig. 4). When comparing the median product of achieved cycles and force as a measure of energy absorbed until failure, the plate fixation group resisted to 1.57 × 10^6^ N*cycles (range, 1.21 × 10^6^ to 2.14 × 10^6^) and the screw fixation group to 3.17 × 10^6^ N*cycles (range, 2.92 × 10^6^ to 3.17 × 10^6^; *p* = .001).

All specimens survived the first 2000 cycles of cyclic loading with up to 1050 N. All plate fixation specimens failed within the subsequent 1000 cycles with a maximum load of 1750 N. The screw fixation samples all survived loading up to 2100 N and 3 of 4 samples also survived the highest loading with 2450 N (Fig. 4).

### Acetabular fracture gap

**Fig. 5:**
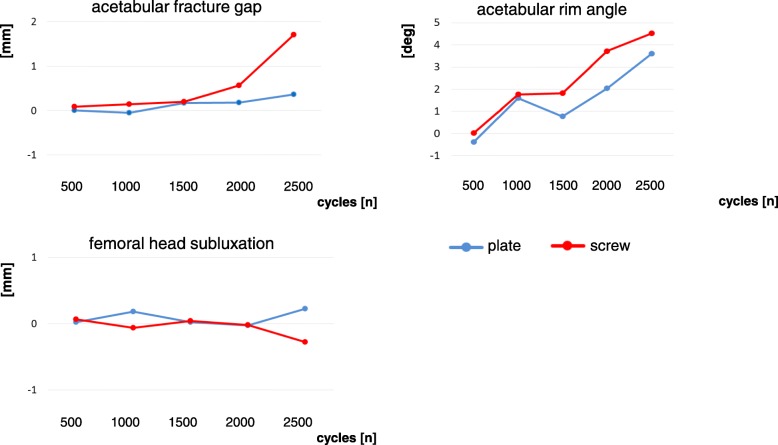
Fracture displacement

The increase in acetabular fracture gap formation under loading with 175 N was not statistically different between the two groups after 500 cycles (*p* = .704), 1000 cycles (*p* = .826), 1500 cycles (*p* = .196), 2000 cycles (*p* = .125), and 2500 cycles (*p* = .244, Fig. 5).

### Acetabular rim angle

The decrease in acetabular rim angle formation was not statistically different between the two groups after 500 (*p* = .740), 1000 (*p* = .824), 1500 (*p* = .834), 2000 (*p* = .151), and 2500 (*p* = .651, Fig. 5).

### Medial femoral head subluxation

The change in femoral head position relative to the acetabular rim was not statistically different at 500 cycles (*p* = .126), 1000 (*p* = .123), 1500 (*p* = .576), 2000 (*p* = .638), and 2500 (*p* = .120, Fig. 5).

### Mode of failure

**Fig. 6:**
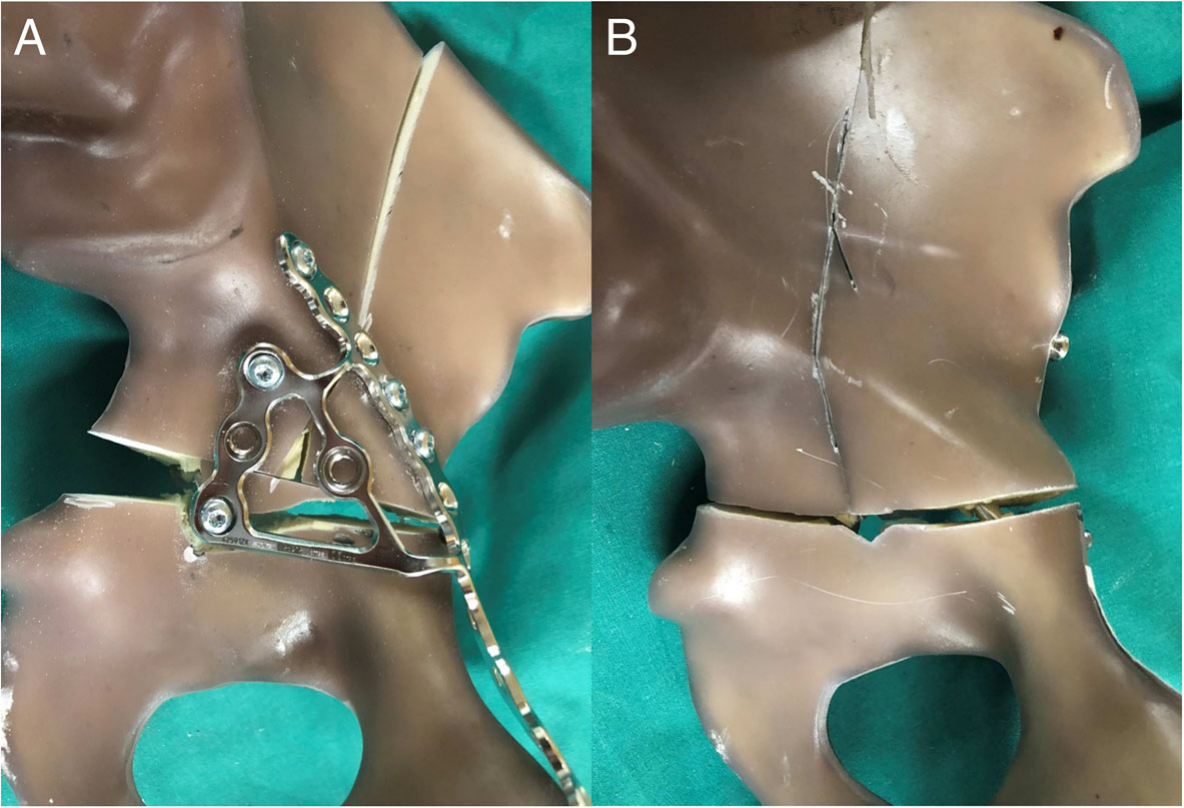
Mode of failure. All plate fixation constructs showed a very similar pattern of failure with a break-out of the posterior-column screw in the buttress-wing of the plate and consecutive opening of the fracture gap (**a**) None of the screw fixation constructs showed a sudden failure in terms of an implant cut-out but rather stepwise implant loosening and widening of the fracture gap (**b**)

With regard to the mode of failure, all plate fixation constructs failed with a break-out of the posterior-column screw in the quadrilateral wing of the anatomical plate. This resulted in an angular posterior opening of the fracture gap and bending of the rim-part of the plate (Fig. 6A). None of the screw fixation constructs showed implant failure or cut-out of the screws. One construct failed by sudden loosening of the screw with an audible crack and widening of the fracture gap (Fig. 6A).

## Discussion

The purpose of this study was to compare the biomechanical strength of lag screw-only fixation versus a standard anatomical buttress plate fixation.

Most studies available investigated different screw and plate fixation constructs and found those to provide high degrees of stiffness and strength in the stabilization of acetabular fractures [16, 22, 23].

In our investigation, we observed superior strength and construct survival of the screw fixation technique. This is in line with findings of a biomechanical study on screw versus plate fixation constructs in iliac crescent fractures [24].

Kistler et al. [16] compared a combination of lag screw and column plates to the same quadrilateral surface buttress plate as used in our study in a very similar experimental setup. Their findings are consistent with our results in the plate fixation group. Before conducting their study in synthetic bone models, they had validated the setup in human cadaver pelvises [16].

The same setup for the testing of acetabular fracture fixation strength was also used by other studies [18, 25]. As, in the authors opinion, fracture fixation failure is a result of repetitive loads and not so much of sudden catastrophic loads, we did not test for load to failure. Instead, the number of cycles was expanded and cyclic loads up to 3.5 body weights were applied in order to approximate the conditions of full weight bearing [26].

This study was an in vitro study using synthetic bone models. The use of synthetic models brings limitations. There is a lack of surrounding soft tissues, which alters the physiological and anatomical behaviour compared to cadaver pelves or the biomechanics in real life. Next to economic and ethical considerations, we chose fourth generation Sawbone models for the advantage of better standardization in construct behaviour. The advantages of using a synthetic model are the homogeneous bone structure and the elimination of individual bone quality as a potential bias [12, 13, 27]. In addition, the pelvic models used in this study had previously been validated against osteoporotic human cadaver bone [16] and, hence, a similar setup has been used by other studies [18, 25]. It would be of interest, though, to repeat these tests with cadaver pelvises in order to assess the influence of different degress of bone quality.

In vitro studies can approximate the effect of fatigue on primary stability but they cannot provide information about the bone’s behaviour through healing and rehabilitation. The samples in this study were loaded to up to 3.5 body weights which may not be realistic in the context of postoperative painful mobilization, although some patients will reach these loads by accident [5].

Acetabular fracture gap, acetabular rim angle, and medial femoral head subluxation were not different between the two groups. However, the patterns of stress distribution may differ noticeably between the two fixation methods tested and the behaviour of acetabular fracture gap, rim angle, and medial femoral head subluxation may not be fully comparable. Construct survival is a result of all these three factors and, in addition, is a measure of the vulnerability of a construct to means of fatigue and accumulated failure. The most relevant finding of this study may not be a potential superiority of the screw fixation technique over a certain type of plate fixation. There are many other ways to use a plate in order to stabilize an acetabulum and the one investigated here may not be the strongest. More importantly, screw-only fixation seems to provide sufficient resistance to the forces that can be expected during immediate full-weight bearing. These in vitro data are in line with the findings in clinical cohorts. Stöckle et al. [28] observed a loss of reduction in only three of 51 fractures during a 2 year follow-up after acetabular fracture fixation with only 3.5 mm screws. Similar results were seen in a small cohort of 13 elderly patients with acetabular fractures treated with lag-screw fixation [11].

Future prospective clinical studies comparing percutaneous versus open fixation of this entity of acetabular fractures may help to identify potential candidates for the less-invasive technique.

## Conclusions

In this in vitro biomechanical study, screw-only fixation of an ACPHT acetabular fracture resulted in at least as good construct survival as seen for standard buttress plate fixation. Both methods resisted sufficiently to forces that would be expected under physiologic conditions. Fixation of ACPHT fractures seems to be a biomechanically valid alternative, where a less invasive treatment is needed.

## Additional file

Additional file 1: Source data. This file provides measurement raw data of the sample testing. (PDF 45 kb)

## Abbreviations

ACPHT: Anterior column and posterior hemi-transverse
LC2: Young & Burgess lateral compression fracture type 2
