# Supplementary material for: Screw fixation of ACPHT acetabular fractures offers sufficient biomechanical stability when compared to standard buttress plate fixation
Source: BMC Musculoskelet Disord. 2019 Jan 24;20:39. doi: 10.1186/s12891-019-2422-6 (PMC6346571; doi:10.1186/s12891-019-2422-6)
Supplement: Supplementary file 1 — Source data. This file provides measurement raw data of the sample testing. (PDF 45 kb) [file 12891_2019_2422_MOESM1_ESM.pdf]

SpecimenNo	Group	Group_coded	Test_Date	cycles_completed	cycles175	cycles350	cycles700
Plate1	PLATE	1	24.02.2018, 14.43	2050	500	500	500
Plate2	PLATE	1	24.02.2018, 18.54/28.02.2018, 9.46	2675	500	500	500
Plate3	PLATE	1	26.01.2018, 10.00/28.01.2018, 10.01	2557	500	500	500
Plate4	PLATE	1	27.02.2018, 14.42	2020	500	500	500
Screw1	SCREW	2	24.02.2018, 8.52	3200	500	500	500
Screw2	SCREW	2	24.02.2018, 15.26/28.02.2018, 9.00	3101	500	500	500
Screw3	SCREW	2	24.02.2018, 20.05/28.02.2018, 9.07	3200	500	500	500
Screw4	SCREW	2	27.02.2018, 9.57/28.02.2018, 9.27	3200	500	500	500

cycles1050	cycles1400	cycles1750	cycles2100	cycles2450	N_cycles	Gap175	Gap175_1	Gap175_2	Gap175_3
500	50	0	0	0	1207500	1,65333	1,63	1,66	1,67
500	500	175	0	0	2143750	2,07667	2,1	2,06	2,07
500	500	57	0	0	1937250	1,26667	1,24	1,26	1,3
500	20	0	0	0	1165500	0,9	0,9	0,86	0,94
500	500	500	100	100	3167500	1,62	1,68	1,6	1,58
500	500	500	100	1	2924950	1,09667	1,06	1,1	1,13
500	500	500	100	100	3167500	0,93667	0,96	0,93	0,92
500	500	500	100	100	3167500	0,18667	0,18	0,18	0,2

GapChange350	Gap350	Gap350_1	Gap350_2	Gap350_3		GapChange700	Gap700	Gap700_1	Gap700_2	Gap700_3
0,28	1,93333333	1,94	1,91	1,95		0,38	2,033333	2	2,04	2,06
0,006666667	2,08333333	2,08	2,08	2,09		0,013333333	2,09	2,09	2,09	2,09
-0,033333333	1,23333333	1,22	1,23	1,25		-0,086666667	1,18	1,2	1,16	1,18
0,083333333	0,98333333	0,99	0,94	1,02		-0,06	0,84	0,84	0,86	0,82
-0,023333333	1,59666667	1,59	1,6	1,6		-0,05	1,57	1,58	1,57	1,56
0,06	1,15666667	1,17	1,18	1,12		0,116666667	1,213333	1,17	1,24	1,23
0,14	1,07666667	1,1	1,07	1,06		0,196666667	1,133333	1,18	1,11	1,11
0,036666667	0,22333333	0,26	0,2	0,21		0,093333333	0,28	0,24	0,29	0,31

GapChange1050	Gap1050	Gap1050_1	Gap1050_2	Gap1050_3		GapChange1400	Gap1400	Gap1400_1	Gap1400_2	Gap1400_3
0,32	1,97333	1,95	1,97	2		0,58	2,233333333	2,2	2,29	2,21
0,17	2,24667	2,24	2,24	2,26		0,176666667	2,253333333	2,25	2,23	2,28
-0,083333333	1,18333	1,17	1,19	1,19		-0,053333333	1,213333333	1,22	1,2	1,22
0,016666667	0,91667	0,94	0,91	0,9		0,006666667	0,906666667	0,91	0,9	0,91
0,48	2,1	2,18	2,12	2		0,736666667	2,356666667	2,37	2,34	2,36
0,21	1,30667	1,3	1,34	1,28		0,196666667	1,293333333	1,3	1,28	1,3
0,266666667	1,20333	1,21	1,23	1,17		0,703333333	1,64	1,68	1,61	1,63
0,136666667	0,32333	0,3	0,31	0,36		0,433333333	0,62	0,5	0,68	0,68

GapChange1750	Gap1750	Gap1750_1	Gap1750_2	Gap1750_3	Gap2100_1	Gap2100_2	Gap2100_3	FemurIn175
0,623333333	2,276666667	2,28	2,29	2,26	3,91	3,25	3,5	5,18
0,216666667	2,293333333	2,29	2,29	2,3	2,99	2,96	3,02	6,61
-0,03	1,236666667	1,24	1,23	1,24	1,23	1,4	1,39	6,46333333
0,786666667	1,686666667	1,73	1,65	1,68	2,15	2,13	2,14	7,15333333
1,023333333	2,643333333	2,65	2,65	2,63	3,67	3,77	3,65	5,28666667
0,366666667	1,463333333	1,45	1,46	1,48	1,99	1,73	1,86	5,41
2,923333333	3,86	3,61	4	3,97	4,16	4,1	4,16	5,54
0,49	0,676666667	0,67	0,7	0,66	1,5	1,47	1,44	4,20333333

FemurIn175_1	FemurIn175_2	FemurIn175_3	FemurChange35	FemurIn350	FemurIn350_1	FemurIn350_2	FemurIn350_3
5,18	5,13	5,23	0,743333333	4,436666667	4,42	4,47	4,42
6,59	6,62	6,62	0,31	6,3	6,37	6,27	6,26
6,43	6,49	6,47	0,113333333	6,35	6,38	6,33	6,34
7,12	7,18	7,16	0,026666667	7,126666667	7,08	7,16	7,14
5,29	5,3	5,27	0,023333333	5,263333333	5,3	5,25	5,24
5,41	5,4	5,42	-0,496666667	5,906666667	5,94	5,91	5,87
5,53	5,56	5,53	0,18	5,36	5,35	5,36	5,37
4,25	4,16	4,2	-0,046666667	4,25	4,27	4,26	4,22

FemurChange700	FemurIn700	FemurIn700_1	FemurIn700_2	FemurIn700_3	FemurChange1050	FemurIn1050	FemurIn1050_1
0,743333333	4,436666667	4,5	4,34	4,47	0,963333333	4,216666667	4,22
0,243333333	6,366666667	6,4	6,39	6,31	0,12	6,49	6,52
0,183333333	6,28	6,3	6,27	6,27	0,026666667	6,436666667	6,4
0,113333333	7,04	7,06	7,02	7,04	0,01	7,143333333	7,12
0,303333333	4,983333333	4,99	4,97	4,99	0,643333333	4,643333333	4,66
-0,393333333	5,803333333	5,78	5,87	5,76	-0,336666667	5,746666667	5,7
0,066666667	5,473333333	5,5	5,48	5,44	0,083333333	5,456666667	5,46
-0,193333333	4,396666667	4,4	4,4	4,39	0,006666667	4,196666667	4,2

FemurIn1050_2	FemurIn1050_3	FemurChange1	FemurIn1400	FemurIn1400_1	FemurIn1400_2	FemurIn1400_3	FemurChange1750	FemurIn1750
4,18	4,25	0,96	4,22	4,23	4,2	4,23	0,92	4,26
6,51	6,44	-0,02666667	6,63666667	6,61	6,66	6,64	0,54	6,07
6,47	6,44	0,023333333	6,44	6,44	6,46	6,42	0,226666667	6,236666667
7,1	7,21	-0,04	7,19333333	7,21	7,17	7,2	0,193333333	6,96
4,64	4,63	0,603333333	4,68333333	4,69	4,69	4,67	0,573333333	4,713333333
5,76	5,78	-0,26666667	5,67666667	5,66	5,73	5,64	-0,21	5,62
5,5	5,41	0,066666667	5,47333333	5,45	5,49	5,48	-0,52	6,06
4,22	4,17	-0,09666667	4,3	4,3	4,33	4,27	-0,033333333	4,236666667

FemurIn1750_1	FemurIn1750_2	FemurIn1750_3	FemurIn2100_1	FemurIn2100_2	FemurIn2100_3	RimAngle175	RimAngle175_1	RimAngle175_2
4,27	4,26	4,25	5,12	4,99	4,86	170,93	171,69	170,17
6,09	6,12	6	5,88	5,83	5,82	169,06	169,51	168,2
6,22	6,27	6,22	6,83	6,86	6,83	175,263333	175,41	175,48
7	6,95	6,93	7	6,98	6,98	165,49	166,22	165,09
4,7	4,73	4,71	5,45	5,24	5,28	171,3	171,12	171,53
5,59	5,64	5,63	6,8	6,8	6,85	173,876667	171,71	176,84
6,02	6,11	6,05	5,7	5,72	5,7	171,606667	172,32	171,06
4,26	4,24	4,21	4,64	4,67	4,69	178,553333	178,7	179,29

RimAngle175_3	RimChange350	RimAngle350	RimAngle350_1	RimAngle350_2	RimAngle350_3	RimChange700	RimAngle700
170,93	0,153333333	170,7766667	170,78	170,09	171,46	-0,32	171,25
169,47	3,83	165,23	164,17	165,98	165,54	8,533333333	160,5266667
174,9	-0,73	175,9933333	175,75	175,72	176,51	1,596666667	173,6666667
165,16	-0,393333333	165,8833333	166,54	165,16	165,95	-1,026666667	166,5166667
171,25	-0,743333333	172,0433333	171,19	172,07	172,87	1,006666667	170,2933333
173,08	1,91	171,9666667	173,58	171,63	170,69	2,213333333	171,6633333
171,44	0,123333333	171,4833333	171,94	171,28	171,23	1,676666667	169,93
177,67	-0,096666667	178,65	178,6	178,92	178,43	1,84	176,7133333

RimAngle700_1	RimAngle700_2	RimAngle700_3	RimChange1050	RimAngle105(	RimAngle1050_1	RimAngle1050_2	RimAngle1050_3
171,42	170,89	171,44	-1,22	172,15	172,72	172,48	171,25
160,9	159,79	160,89	7,94	161,12	161,88	161,47	160,01
174,09	173,47	173,44	0,426666667	174,836667	174,44	175,65	174,42
168,44	165,95	165,16	0,77	164,72	165,69	164,32	164,15
171,29	169,94	169,65	0,346666667	170,953333	171,29	170,75	170,82
171,44	171,37	172,18	2,13	171,746667	169,82	173,68	171,74
168,28	170,46	171,05	1,426666667	170,18	171,99	168,79	169,76
177,21	176,48	176,45	2,193333333	176,36	176,42	176,27	176,39

RimChange1400	RimAngle140(	RimAngle1400_1	RimAngle1400_	RimAngle1400_3	RimChange1750	RimAngle1750	RimAngle1750_1
-0,876666667	171,806667	171,43	171,61	172,38	-0,686666667	171,6166667	171,67
3,833333333	165,226667	165,17	165,23	165,28	5,23	163,83	163,98
1,006666667	174,256667	173,6	174,54	174,63	4,763333333	170,5	171,49
-0,27	165,76	164,03	164,7	168,55	3,163333333	162,3266667	163,44
2,04	169,26	168,89	170,85	168,04	3,15	168,15	167,76
3,14	170,736667	170,19	171,35	170,67	3,603333333	170,2733333	171,56
1,846666667	169,76	171,12	168,37	169,79	1,92	169,6866667	171,09
5,59	172,963333	172,23	173,19	173,47	7,12	171,4333333	171,37

RimAngle1750_2	RimAngle1750_3	RimAngle2100_1	RimAngle2100_2	RimAngle210(
171,05	172,13	170,58	169,74	171,01
163,77	163,74	164,23	163,67	163,92
169,28	170,73	169,09	171,41	169,91
160,92	162,62	155,67	160,14	156,75
168,93	167,76	169,71	169,6	170,56
169,08	170,18	168,31	169,76	168,64
169,05	168,92	170,03	169,62	168,02
170,96	171,97	171,37	171,56	171,26
